# Generation of Functional Blood Vessels from a Single c-kit+ Adult Vascular Endothelial Stem Cell

**DOI:** 10.1371/journal.pbio.1001407

**Published:** 2012-10-16

**Authors:** Shentong Fang, Jing Wei, Nalle Pentinmikko, Hannele Leinonen, Petri Salven

**Affiliations:** 1 Department of Pathology, University of Helsinki, Helsinki, Finland; 2 Haartman Institute, University of Helsinki, Helsinki, Finland; 3 Research Programs, University of Helsinki, Helsinki, Finland; Baylor College of Medicine, United States of America

## Abstract

In adults, the growth of blood vessels, a process known as angiogenesis, is essential for organ growth and repair. In many disorders including cancer, angiogenesis becomes excessive. The cellular origin of new vascular endothelial cells (ECs) during blood vessel growth in angiogenic situations has remained unknown. Here, we provide evidence for adult vascular endothelial stem cells (VESCs) that reside in the blood vessel wall endothelium. VESCs constitute a small subpopulation within CD117+ (c-kit+) ECs capable of undergoing clonal expansion while other ECs have a very limited proliferative capacity. Isolated VESCs can produce tens of millions of endothelial daughter cells in vitro. A single transplanted c-kit-expressing VESC by the phenotype lin−CD31+CD105+Sca1+CD117+ can generate in vivo functional blood vessels that connect to host circulation. VESCs also have long-term self-renewal capacity, a defining functional property of adult stem cells. To provide functional verification on the role of c-kit in VESCs, we show that a genetic deficit in endothelial c-kit expression markedly decreases total colony-forming VESCs. In vivo, c-kit expression deficit resulted in impaired EC proliferation and angiogenesis and retardation of tumor growth. Isolated VESCs could be used in cell-based therapies for cardiovascular repair to restore tissue vascularization after ischemic events. VESCs also provide a novel cellular target to block pathological angiogenesis and cancer growth.

## Abstract 2

Adult vascular endothelial stem cells are shown to reside in the blood vessel wall endothelium. When isolated, these cells are capable of clonal expansion and generate functional blood vessels in vivo.

## Author Summary

Angiogenesis—the growth of blood vessels—is essential for organ growth and repair, but also occurs during tumorigenesis and in certain inflammatory disorders. All blood vessels are lined by endothelial cells (ECs)—thin, flattened cells that form a continuous monolayer throughout the entire circulatory system. The cellular origin of new vascular ECs during blood vessel growth in angiogenic situations in adults is a matter of debate. New ECs could develop, in principle, from as yet undiscovered stem cells, as is well documented for the differentiated cells of skin or epithelia, or by the duplication of existing differentiated ECs. Here, we provide evidence for the existence of vascular endothelial stem cells (VESCs) that reside in the adult blood vessel wall endothelium. VESCs constitute a small subpopulation of ECs capable of clonal expansion, while other ECs have a very limited proliferative capacity. When isolated, these VESCs can produce tens of millions of endothelial daughter cells, and a single transplanted VESC can generate in vivo functional blood vessels that connect to host blood circulation. Isolated VESCs could be used in cell-based therapies for cardiovascular repair to restore tissue vascularization following ischemia and could also be pursued as a novel cellular target of inhibition to block pathological angiogenesis, for example during tumor growth.

## Introduction

The early blood vessels of the embryo and yolk sac in mammals develop by aggregation of de-novo-forming angioblasts into a primitive vascular plexus (vasculogenesis). Blood vessels arise from endothelial precursors, which share an origin with hematopoietic progenitors [1]–[3]. In adults, the growth of blood vessels is essential for organ growth and repair. The best-known pathological conditions in which angiogenesis is switched on are malignant [4], ocular, and inflammatory disorders [5]. Endothelial cells (ECs) are thin, flattened cells that line the inside of blood vessels in a continuous monolayer in all blood vessels through the entire circulatory system. ECs are best identified by their specific location and function, but there are also various cell-surface molecules (such as vWF, CD31, CD34, CD105, vascular endothelial cadherin [VE-cadherin], vascular endothelial growth factor receptor 1 [VEGFR-1], VEGFR-2, Tie-1, Tie-2) that characterize their phenotype [6],[7]. Recently, Weissman and coworkers by performing genetic fate mapping and clonal analysis of individual cells showed that the endothelial stem/progenitor cells involved in adult angiogenesis must be local, non-hematopoietic, and non-circulating tissue resident cells [8]. However, the definite cellular origin of the new ECs necessary for adult neoangiogenesis has remained unknown [8]–[16]. Creation of new ECs in adult tissues could in principle occur by their so far undiscovered tissue resident stem cells, as is well documented for the differentiated cells of skin or epithelia [17]–[19], or by the duplication of existing differentiated ECs, as has been described for pancreatic beta-cells [20].

In passaged human aortic ECs not all cells in the monolayers proliferate at an equal rate [21]. Previous work has also indicated that very low numbers of cells with endothelial characteristics and high proliferative potential may be found in umbilical cord blood or in peripheral blood [22]–[26]. Together, these earlier findings suggest that all ECs in adult tissues may not have an equal potential to produce progeny. Therefore, we wanted to learn if there exists a rare vascular endothelial stem cell (VESC) population that is capable of producing very high numbers of endothelial daughter cells and is responsible for neovascular growth in adults.

## Results

### Adult lin−CD31+CD105+ ECs Encompass Rare Endothelial Colony-Forming Cells

**Figure 1:**
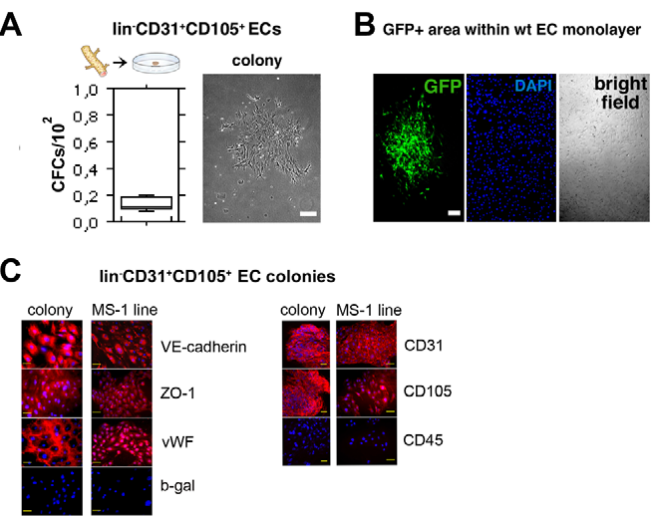
Isolated adult lin−CD31+CD105+ ECs encompass rare endothelial CFCs. (A) Quantification of colony-forming ability of lin−CD31+CD105+ cells isolated from the mouse lungs. Freshly isolated cells from ten mice were assayed in duplicate. An EC colony in the semi-solid matrix is also shown. The colonies grow beneath the methylcellulose matrix adhered to the bottom of the culture dish. Scale bar, 150 µm. (B) Freshly isolated GFP-tagged ECs were seeded on standard 2-D EC cultures one CFU per culture dish together with 20 CFUs of wt ECs and grown until the monolayer was confluent. GFP+ cells form a single circular EC batch within the confluent wt EC monolayer, demonstrating the clonal growth pattern of the ECs responsible for creating the confluent monolayer. DAPI and bright field images show also the wt ECs in the confluent monolayer. Scale bar, 200 µm. (C) Colonies formed from isolated lin−CD31+CD105+ mouse lung ECs in low-cell density adherent semi-solid methylcellulose matrix were stained for various cell-surface markers. The colonies express EC markers CD31, CD105, VE-cadherin, and vWF while the cells are negative for the pan-hematopoietic marker CD45. The nuclei are stained with DAPI (blue) to recognize individual cells. MS-1 murine EC line was used as a control. Rabbit anti-β-gal antibodies and rat anti-mouse CD45 antibodies provide the isotype controls for rabbit and rat antibodies, respectively. Scale bars, 50 µm.

In preliminary experiments we found that rare endothelial colony-forming cells (CFCs) were routinely detected when ECs were isolated from single cell suspensions prepared by enzymatic digestion of adult mouse tissues, and cultured in vitro in low-cell density adherent semi-solid methylcellulose matrix colony assays supplemented with the endothelial growth factor VEGF. To study the possibility that endothelial CFCs might reside in the vascular wall endothelium, we perfused adult C57BL/6J mice with PBS to wash out the circulating hematopoietic cells, and subsequently isolated CD31+CD105+ ECs from the lung vasculature and tested them in vitro in colony assays. After removal of the remaining contaminating hematopoietic cells by immunomagnetic lineage depletion using a standard combination of antibodies designed to remove mature hematopoietic cells from a sample based on their hematopoietic surface-marker (CD5, CD45R [B220], CD11b, Gr-1 [Ly-6G/C], 7-4, and Ter-119) expression, endothelial CFCs could be recovered from the tissues at higher frequencies. In these isolated lineage depleted (lin−) CD31+CD105+ mouse lung ECs, CFCs were detected at a mean frequency of 0.14% (standard deviation [SD]±0.051; *n* = 10), corresponding to approximately one and a half colony-forming units (CFUs) per thousand isolated ECs (Figure 1A). These EC colonies grow beneath the methylcellulose matrix adhered to the plastic bottom of the culture dish (Figure 1A). The formed colonies express phenotypic EC markers CD31, CD105, VE-cadherin, and vWF while the cells are negative for the pan-hematopoietic marker CD45 (Figure 1C).

To further study in vitro the endothelial CFCs, we isolated CD31+CD105+ ECs from transgenic C57BL/6-Tg(ACTB-EGFP)1Osb/J mice where all the tissues, including the ECs, are green fluorescent protein (GFP)+. Subsequently, we seeded a mixture of one CFU of GFP+ CD31+CD105+ ECs and 20 CFUs of wild-type (wt) CD31+CD105+ ECs on a standard 2-D EC culture and let the cells grow until the monolayer was confluent, typically for 12 d. The resulting confluent monolayers of wt ECs contained on average one circular, GFP+ EC batch per culture, demonstrating the clonal growth pattern of the ECs responsible for creating the confluent monolayer (Figure 1B).

### Colony-Forming ECs Produce Tens of Millions of Endothelial Daughter Cells In Vitro

**Figure 2:**
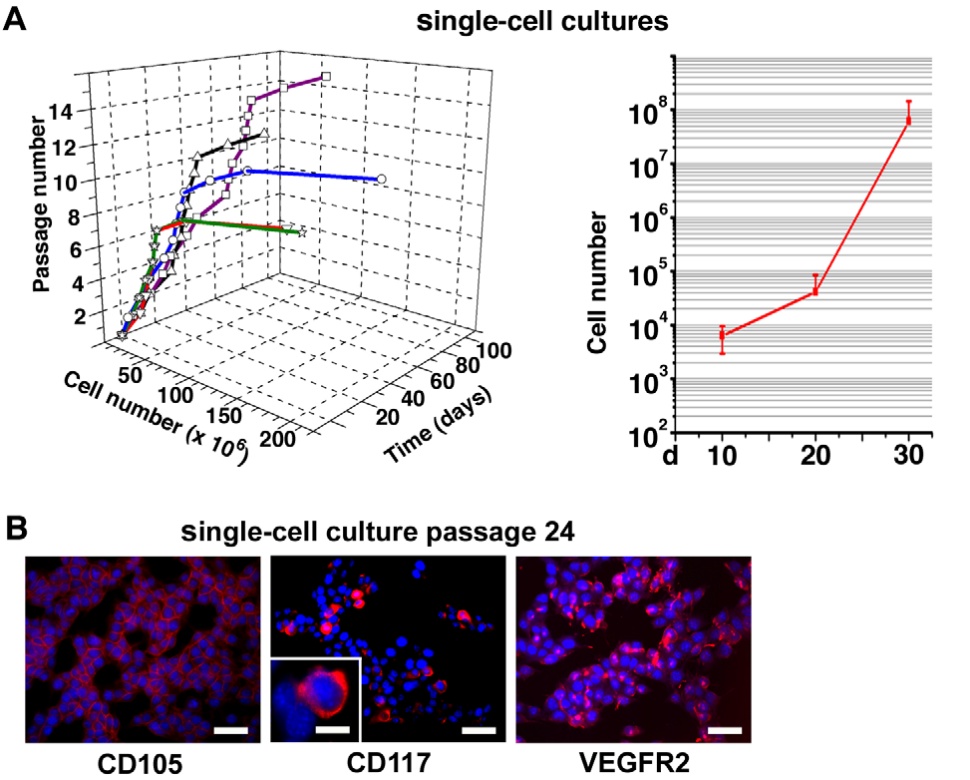
Isolated colony-forming ECs produce tens of millions of endothelial daughter cells in vitro. (A) Growth kinetics of five separate EC monolayer cultures originating from individual lin−CD31+CD105+ CFCs that were picked up from colony assays and propagated in 2-D cultures. The cultures were split when 90% confluent and no cells were discarded during the experiment. A summary growth curve (mean ± SD) of the five cultures is also shown. Cell number is in log scale. (B) The long term 2-D EC cultures were analyzed at passage 24 by immunofluorescence microscopy. The cells express the endothelial markers CD105, VEGFR-2, and the stem/progenitor cell marker CD117. The nuclei are stained with DAPI (blue) to recognize individual cells. Scale bars, 50 µm; 5 µm (insert).

To assess the proliferative potential of the CFCs in vitro, we picked up individual colonies, resuspended them in EC growth medium, and cultured the cells as monolayers in 2-D EC cultures. Some of the formed monolayers were propagated for over 6 mo, splitting the cultures always when they reached 90% confluence (Figure 2). The cultures were transferred in large 75 cm^2^ flasks after an average of ten passages (Figure S1B). When a total of 148 separate colonies originating from distinct CFCs were picked up and cultured ex vivo as monolayers, a total of five colonies could be expanded and passaged for over ten generations, and three of them were propagated for over 20 passages. During the long-term culture, each of these five colonies originating from a single CFC produced tens of millions of daughter cells (Figure 2A). The long-term cultured colony cells express EC markers CD105, VEGFR-2, and the stem/progenitor cell marker CD117 (Figure 2B). Surface marker expression of EC monolayers originating from single CFCs is summarized in Table S1, and micrographs of immunostainings against the cell-surface markers at the 24th passage are shown in Figure S1. Taken together, the experiments demonstrate that a subpopulation of colony-forming ECs exists that are capable of clonal expansion and can produce tens of millions of endothelial daughter cells when expanded as EC monolayers ex vivo.

### CD117+ EC Population Enriches for Rare Endothelial CFCs

**Figure 3:**
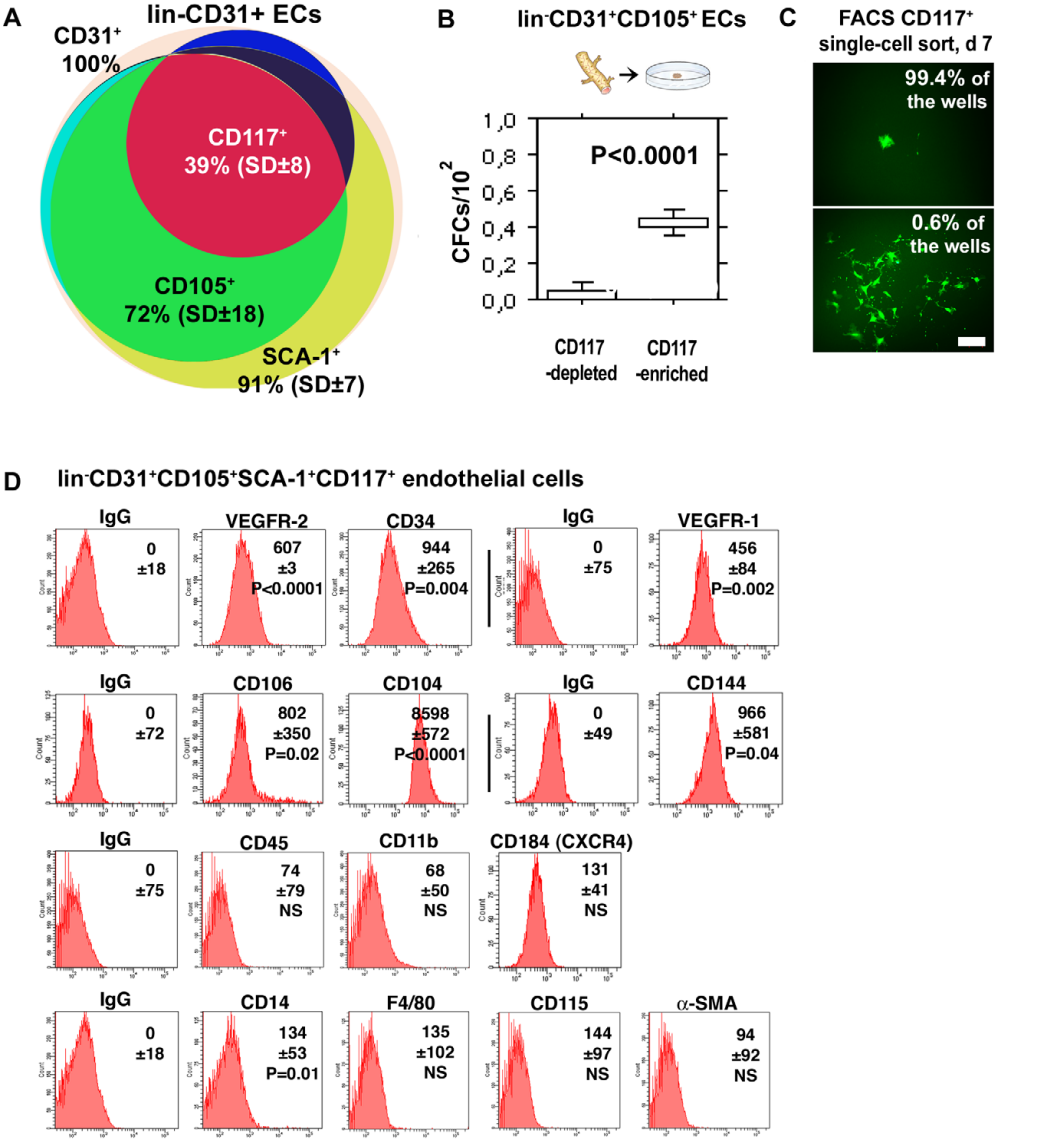
The CD117+ EC population enriches for rare adult colony-forming ECs. (A) The relative distributions and overlap of CD105+, Sca-1+, and CD117+ subpopulations within isolated mouse lung lin−CD31+ ECs are shown. The results (mean ± SD) are from six FACS analyses of six mice. (B) Comparison of CFCs within the CD117-depleted and CD117-enriched fractions of lin−CD31+CD105+ ECs in vitro in colony-forming assays. Practically all CFCs are encompassed within the CD117+ EC population (*p*<0.0001, the Mann-Whitney test). The horizontal lines indicate 10th, 25th, 50th (median), 75th, and 90th percentiles. The results of four independent experiments, each performed in duplicate, are shown. (C) A total of 960 freshly isolated GFP+ lin−CD31+CD105+Sca1+CD117+ single cells were sorted into individual wells of 96-well plates together with a carrier population of 2,500 wt (GFP−) lin−CD31+CD105+ cells per well. At day 7, most (99.4%) of the generated EC monolayers contained only a few GFP+ ECs (above). On six wells (0.6%), the generated monolayer contained a circular, clonal area of GFP+ ECs within the otherwise GFP− monolayer (below). The result thus again reveals a small subpopulation within CD117+ ECs that are capable of undergoing clonal expansion while the other ECs have a very limited proliferative capacity. Scale bar, 200 µm. (D) Freshly isolated murine lung lin−CD31+CD105+Sca1+CD117+ ECs were assayed for a large set of endothelial, hematopoietic, and smooth muscle cell-surface markers using FACS. The results show that isolated lin−CD31+CD105+Sca1+CD117+ are highly immunoreactive for various endothelial markers while no immunoreactivity is detected against specific hematopoietic markers or against smooth muscle α-actin (SMA). The numbers indicate the mean fluorescence intensity (MFI) values (±SD) of the gated populations calculated from three mice over three experiments, and the corresponding *p*-values tested for significance in comparison to the respective IgG control. *p*-Values<0.05 were considered significant (Student's *t* test).

To further characterize the colony-forming ECs we analyzed their frequency in various subpopulations of lin− ECs isolated from enzymatically digested mouse lung vasculature using fluorescence activated cell sorter (FACS). Sorting against endothelial-specific markers CD31 and CD105 and against CD117 and Sca-1, molecules that are expressed by many adult stem cell types including hematopoietic stem cells (HSCs) and prostate and mammary gland stem cells [27]–[30], was utilized first. CD117 (c-kit, stem cell growth factor receptor [SCFR]) plays an important role in adult HSCs survival and proliferation [27]. Sca-1 is a GPI-anchored cell surface protein that plays a role in modulating CD117 expression and characterizes mouse bone marrow (BM) subset that contains pluripotent HSCs [31]. In addition to certain adult stem cell types, CD117 and Sca-1 are also expressed by some differentiated adult cells. These include mast cells, dendritic cell subsets, and melanocytes (CD117) [32]–[34], and activated T cells (Sca-1) [32],[35]. We found that while CD105 and Sca-1 were expressed by most lin−CD31+ ECs in the lung, CD117+ ECs represent a more infrequent EC subpopulation (39% of all lin−CD31+ lung ECs) (Figure 3A). We therefore prepared CD117-enriched and CD117-depleted fractions of isolated lin−CD31+CD105+ ECs, and assayed them in vitro for CFCs. The CD117-enriched fraction contained CFCs with an almost 10-fold frequency (0.42%, SD ± 0.054 versus 0.045%, SD±0.037; *p*<0.0001) (Figure 3B) compared to CD117-depleted ECs that contained only few CFCs.

To estimate the frequency of endothelial CD117+ CFCs in yet another assay, a total of 960 freshly isolated GFP+ lin−CD31+CD105+Sca-1+CD117+ single cells were sorted into individual wells of 96-well plates together with a carrier population of lin−CD31+CD105+ wt (GFP-negative) ECs. The presence of a single GFP+ cell per well was checked under fluorescence microscope after sorting. In 7 d, the wt EC carrier population formed a wt (GFP−) EC monolayer in the wells, and the contribution of the single GFP+ cell to the EC monolayer could be studied. The wt (GFP−) EC monolayers on six of the plated 960 wells (0.6% of all wells) contained a clonal area of more than 20 GFP+ ECs (Figure 3C). The rest of the wells (99.4%) contained only a few GFP+ ECs at most (Figure 3C). This occurrence frequency for CD117+ colony-forming ECs (0.6%) is well in line with the results we obtained using the methylcellulose matrix colony assays (Figure 3B).

Further cell-surface marker analyses were performed to better characterize the isolated CD117+ EC population that encloses the progenitor ECs responsible for the clonal expansion. Isolated lin−CD31+CD105+Sca1+CD117+ cells were found to be highly immunoreactive for various established endothelial-cell markers including VE-cadherin (CD144), vascular cell adhesion molecule 1 (VCAM-1, CD106), VEGFR-2, VEGFR-1, CD104 (integrin beta 4), CD34, and for CD14, a marker that is expressed both by hematopoietic cells and by ECs [36]–[39]. No immunoreactivity was detected against hematopoietic lineage markers such as CD45 (leukocyte common antigen), CD11b (macrophage-1 antigen, Mac-1), CXCR4 (chemokine receptor type 4, CD184), F4/80 (a pan macrophage marker), CD115 (macrophage colony-stimulating factor receptor), or against smooth muscle α-actin (Figure 3D). mRNA expression profile from the isolated ECs was analyzed by using real-time quantitative reverse transcription (RT)-PCR, and corresponded to what is expected from ECs (Figure S2). Taken together, the results provide functional evidence that CD117+ ECs are enriched for endothelial CFCs, and that there exists a small subpopulation within CD117+ ECs that are capable of undergoing clonal expansion while other ECs have a very limited proliferative capacity.

### Quiescent Vascular Endothelium as well as Growing Neoangiogenic and Tumor Vessels Contain CD117+ ECs

**Figure 4:**
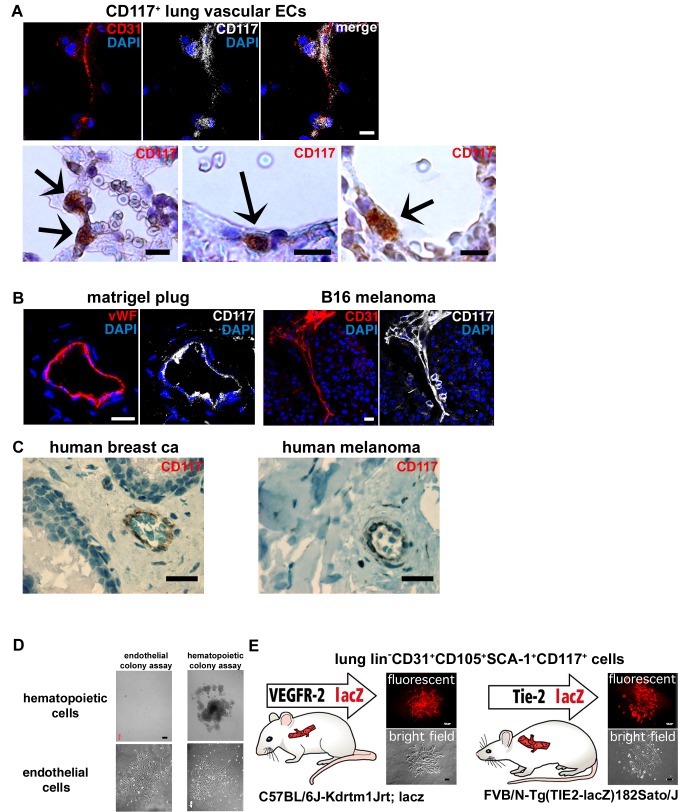
Quiescent vascular endothelium as well as growing neoangiogenic and tumor vessels contain a subpopulation of CD117+ ECs. (A) CD117+ cells localize in the vascular endothelium. High resolution confocal scans from CD31/CD117/DAPI co-staining of mouse lung capillaries are shown. Bright field CD117 immunohistochemistry stainings are also shown. Note the red blood cells at the vessel lumina that is located adjacent to the CD117+ ECs. Scale bars, 10 µm. (B) Neoangiogenic vessels within subcutaneous matrigel plugs and B16 melanoma tumors contain numerous or CD117+ (white) ECs. ECs are stained for CD31 or vWF (both red). High resolution confocal scans are shown. Scale bars, 20 µm. (C) CD117+ ECs are detected also in the tumor vasculature in human malignant melanomas and in human breast cancer. Scale bars, 25 µm. (D) The possibility that contaminating hematopoietic stem or progenitor cells are the origin of the EC colonies was studied in control experiments. No colonies were formed by isolated BM lin− cells when studied in endothelial colony-forming assays (six independent experiments performed in duplicate). BM lin− cells were separated by standard immunomagnetic lineage depletion. In standardized hematopoietic colony-forming assays BM lin− cells produced classical hematopoietic colonies, confirming their viability. Lung lin−CD31+CD105+Sca1+CD117+ ECs formed only EC colonies in both assay formats. Scale bars, 200 µm. (E) Two different genetic reporter systems for the gene expression of VEGFR-2 and Tie-2, receptor tyrosine kinases that are expressed by vascular ECs were used to further test the endothelial origin of the isolated CFCs. lin−CD31+CD105+Sca1+CD117+ cells were isolated from the transgenic mice and then analyzed for the activity of the lacZ-β-gal reporter system (in red fluorescence) in the formed endothelial colonies. Fluorescence and bright field channel images of the colonies are shown. Colonies from the VEGFR-2 promoter lacZ mice and from the Tie-2 lacZ mice express the reporter gene (red fluorescence). Scale bars, 200 µm.

lin−CD31+CD105+Sca-1+CD117+ ECs were detected in various different tissues including subcutaneous tissues, lung, liver, and kidney. In the liver, these CD117+ ECs comprised 18% (mean; SD±12; *n* = 5) of all lin−CD31+ ECs, while in the kidney they were observed more infrequently (mean 2%; SD±1; *n* = 4). CD117+ ECs were detected in capillaries as well as in arteries and veins (Figures 4A and S3). Abundant CD117+ ECs were discovered in neoangiogenic vessels in subcutaneous matrigel plugs and in B16 melanoma tumors (Figure 4B). CD117+ ECs were also detected in tumor vasculature of all randomly picked human cancer samples that were analyzed (Figure 4C; human malignant melanomas and invasive breast cancers, *n* = 14).

The possibility that hematopoietic lineage cells might constitute the EC CFCs was further studied using lineage depleted or CD45-enriched murine BM hematopoietic cells. In standardized murine hematopoietic CFC assays the BM hematopoietic produced classical hematopoietic colonies thus confirming their viability. In contrast, lin−CD31+CD105+Sca1+CD117+ lung ECs produced only completely dissimilar EC colonies on the same assay system (Figures 4D and S4). Importantly, the hematopoietic lin− or CD45+ BM cells could not form any colonies in the endothelial colony assay format even when plated up to 100,000 cells per plate (Figure 4D; for both populations six independent experiments were performed in duplicate). Therefore, we conclude the EC colonies we have studied here do not originate from hematopoietic cells.

Additionally, we also tested our findings by using two different genetic reporter systems for the gene expression of the receptor tyrosine kinases VEGFR-2 and Tie-2 (Figure 4E). These transgenic reporter systems were chosen because VEGFR-2 and Tie-2 are expressed primarily by vascular ECs (although also VEGFR-2+ or Tie-2+ HSCs have been described). lin−CD31+CD105+Sca1+CD117+ cells were isolated from transgenic mice with a lacZ-β-gal reporter under the VEGFR-2 promoter (C57BL/6J-Kdrtm1Jrt; lacZ mice [40]) or under the Tie-2 promoter (FVB/N-Tg(TIE2-lacZ)182Sato/J mice [41]). The isolated lin−CD31+CD105+Sca1+CD117+ cells were then studied in colony assays and analyzed for the activity of the lacZ-β-gal reporter system using fluorescence-based detection of β-galactosidase activity. The colonies both from the VEGFR-2 promoter mice and from the Tie-2 promoter mice promptly expressed the β-gal reporter thus confirming the corresponding marker gene promoter activity (Figures 4E and S4C). Taken together, we conclude that all our data collectively indicate that the EC colonies we observed here originate from vessel wall lin−CD31+CD105+Sca1+CD117+ ECs and are not produced by possible contaminating hematopoietic stem or progenitor cells. These data are also in agreement with recent results by Weissman and coworkers, which by genetic fate mapping and clonal analysis of individual cells demonstrate that endothelial stem/progenitor cells involved in adult angiogenesis must be local, non-hematopoietic, and non-circulating tissue resident cells [8].

### A Single c-kit-Expressing Adult Stem Cell with the Phenotype lin−CD31+CD105+Sca-1+CD117+ Can Generate Functional Blood Vessels In Vivo

**Figure 5:**
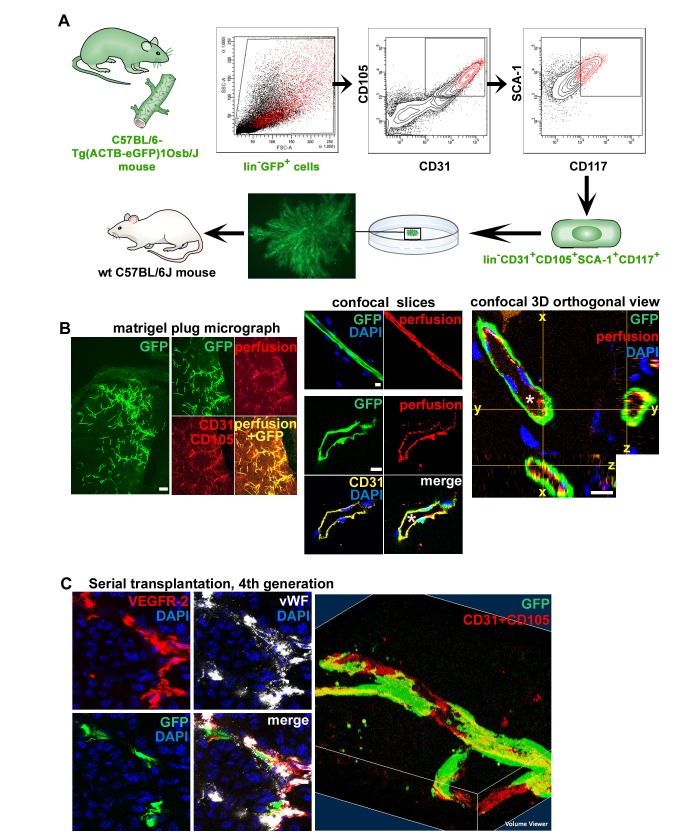
A single c-kit-expressing adult VESC by the phenotype lin−CD31+CD105+Sca-1+CD117+ can generate functional, perfused blood vessels in vivo. (A) Flow diagram of the FACS sorting procedure used to obtain lin−CD31+CD105+Sca1+ CD117+ cells is shown. A single clonal colony originating from a single GFP-tagged lin−CD31+CD105+Sca1+CD117+ CFC was expanded for 12 d in adherent culture to amplify the cell number, manually picked up using a micropipette, mixed with 200 µl of matrigel supplemented with VEGF and bFGF, and injected subcutaneously into wt C57BL/6J mice. An eGFP channel inverted microscope micrograph of a single colony prior that was picked up and transplanted to a wt host is also shown. Scale bar, 150 µm. (B) Functional, perfused GFP+ blood vessels generated by the transplanted descendants of a single c-kit-expressing colony-forming EC by the phenotype lin− CD31+CD105+Sca1+CD117+ cell (14 d after transplantation). The mouse was perfused with fluorescent 0.2 µm microspheres (red) to stain endothelia in functional blood vessels that are connected to the blood circulation. ECs were stained for CD31 or CD105. Scale bars, 100 µm. 1-µm thick confocal optical slices and a 3-D orthogonal projection (x–z and y–z axes) are also shown. Note the blood vessel lumina (*) and the red endothelial signal from the microsphere perfusion of functional vasculature. Scale bars, 10 µm. Six independent experiments with similar results were performed. (C) Self-renewal capacity, a defining characteristic of stem cells, was evaluated by inoculating mice with syngeneic B16 melanomas (2 million cells per mice) together with 15 CFUs of GFP-tagged isolated CD31+CD105+ ECs. After 2 wk of tumor growth, repeated isolations and serial transplantations of lineage depleted single cell suspensions containing the GFP+ tagged ECs and the B16 cells were performed. The figure shows GFP+ blood vessels in the quaternary transplant. ECs were stained for VEGFR-2 (red), vWF (white), and CD31 and CD105 (red). Scale bar, 10 µm. A 3-D reconstitution of a GFP+ blood vessel in the quaternary transplant is also shown (right; a 34-µm thick stack of 34 x–y slices from a confocal scan). Six independent experiments with similar results were performed.

To learn if entire blood vessels could originate from one single c-kit-expressing EC, we performed in vivo transplantations of GFP-tagged ECs originating from a single lin−CD31+CD105+Sca1+CD117+ CFC (Figure 5A). GFP-tagged lin−CD31+CD105+Sca1+CD117+ cells were first isolated from transgenic C57BL/6-Tg(ACTB-EGFP)1Osb/J mice, and the single cell suspension was plated in adherent colony-forming assays at one CFC per plate and cultured for 12–14 d. The plates that later contained only a single clonal colony were utilized in cell transplantations. The single colonies were manually picked up using a micropipette and an inverted microscope, resuspended, and transplanted in matrigel into wt C57BL/6J mice. When the matrigel plugs were analyzed 14 d later, we observed GFP-expressing CD31+ CD105+ blood vessels (Figure 5B). Perfusion of the mice with fluorescein-labeled microspheres, a standard technique to visualize endothelia of functional blood vessels [42],[43], revealed that the GFP+ vessels generated by the transplanted descendants of a single lin−CD31+CD105+Sca1+CD117+ cell were functional blood vessels connected to the host blood circulation (Figure 5B).

Self-renewal is a defining functional property of adult stem cells, which therefore have the ability to repeatedly respond to tissue injury or other growth stimulus by giving rise to substantial numbers of proliferative progenitors. To determine whether the endothelial CFCs would retain their capacity to generate functional blood vessels, we performed serial transplantations with GFP-tagged ECs. We first inoculated C57BL/6J mice with B16 melanoma tumors mixed with 15 CFUs of GFP-tagged CD31+CD105+ ECs, and performed repeated isolations and serial transplantations of lineage depleted single cell suspensions from the tumors every time after 2 wk of tumor growth. GFP+ blood vessels were observed in secondary, tertiary, and quaternary transplants (Figure 5C). These findings provide direct evidence that the GFP-tagged ECs contained VESCs with self-renewal capacity.

**Figure 6:**
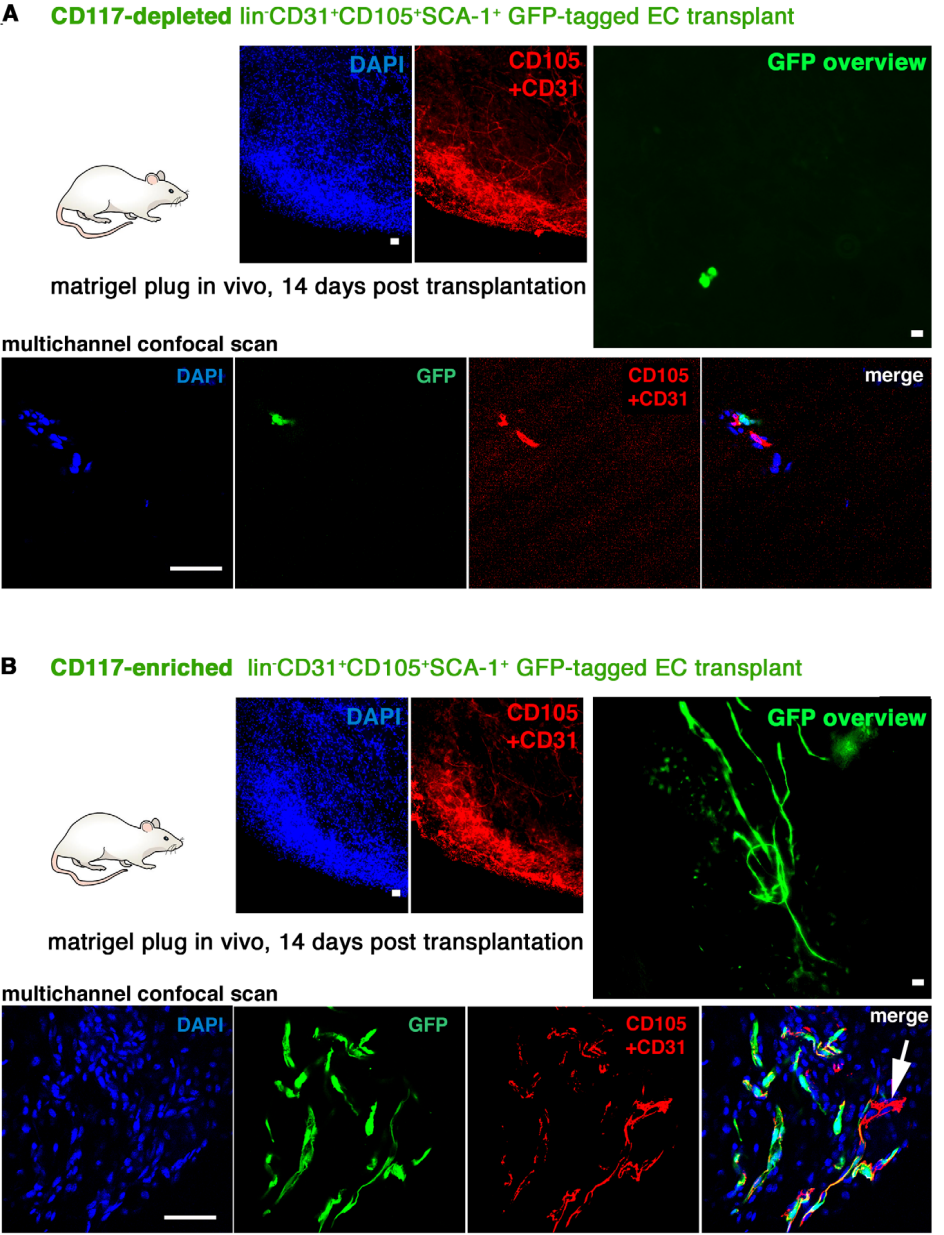
The CD117+ EC population greatly enriches for ECs capable of generating functional blood vessels in vivo. (A) CD117-depleted GFP-tagged lin−CD31+CD105+Sca-1+ ECs were transplanted in matrigel plugs (here 10,000 ECs per plug) into wt C57BL/6 mice. 14 d later, none of the plugs (*n* = 20) contained GFP+ blood vessels. Note that occasional GFP+ ECs from the CD117-depleted GFP+ transplant can be seen within the plug, but they are infrequent (corresponding to the transplanted cell density of 50 ECs per 1 µl) and do not form complete blood vessels. A confocal scan of an area with occasional GFP+ cells is also shown. (B) An equal number (here 10,000) of CD117-enriched GFP+ lin−CD31+CD105+Sca-1+ ECs formed GFP+ blood vessels in all the matrigel plugs (*n* = 12) in an identical control experiment. Overview fluorescence micrographs of the plugs and confocal scans of the sectioned pugs are shown for both groups. Scale bars, 50 µm. Note that in addition to donor GFP+ ECs from the EC transplant the matrigel plugs also contain various host-derived non-EC types such as perivascular pericytes, other mesenchymal/stromal cells, and numerous infiltrating inflammatory cells. Therefore, many cells within a plug do not express endothelial cell markers such as CD31 or CD105. Additionally, the plugs also contain numerous GFP-negative wt ECs and blood vessels from the wt host (arrow).

To study in vivo if the CD117+ EC subpopulation is enriched for endothelial stem and progenitor cells capable of creating blood vessels in adults, we transplanted CD117-depleted or CD117-enriched GFP-tagged lin−CD31+CD105+Sca-1+ ECs in matrigel (10,000 or 100,000 ECs per plug) into wt C57BL/6J mice, and analyzed the plugs 14 d later for the presence of GFP+ blood vessels (Figure 6). None of the plugs in mice transplanted with CD117-depleted ECs (*n* = 20) contained any GFP+ vessels (Figure 6A). Solitary GFP+ ECs from the CD117-depleted transplant were observed within the plugs, but they were infrequent (corresponding to the transplanted EC number) and did not form complete blood vessels. In contrast, all the plugs in mice transplanted with CD117-enriched ECs (*n* = 12) contained numerous GFP+ blood vessels (Figure 6B). Thus, the VESCs with a potential to create novel blood vessels in adults were greatly enriched in the CD117+ EC fraction.

### A Genetic Deficit in Endothelial c-kit Expression Results in Impaired Angiogenesis and Retardation of Tumor Growth

**Figure 7:**
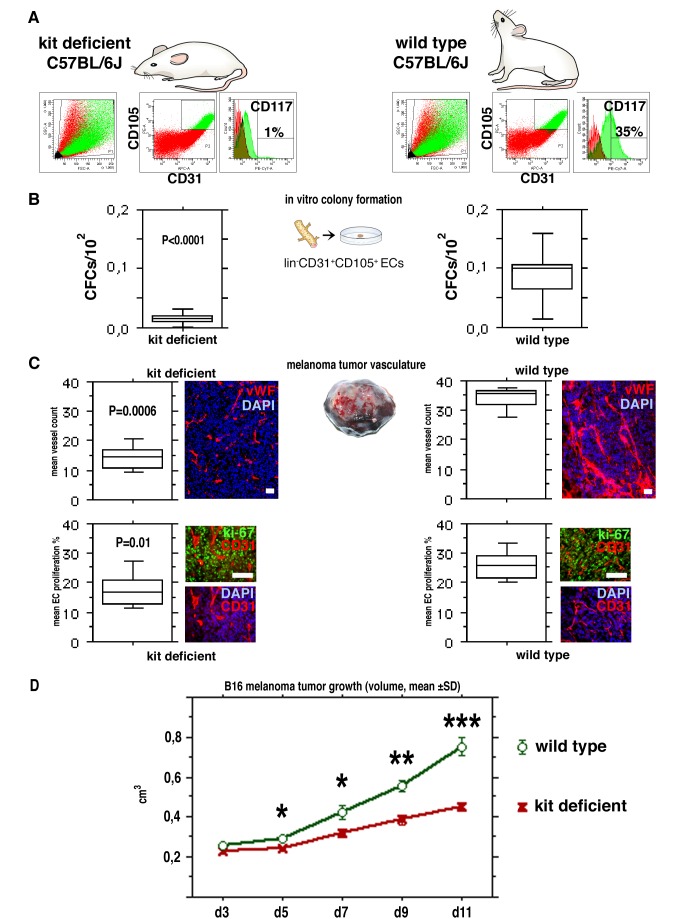
A genetic deficit in endothelial c-kit expression decreases colony-forming VESCs and results in impaired EC proliferation and angiogenesis, and retardation of tumor growth in vivo. (A) Equivalent lin−CD31+CD105+ EC populations were detected in mutant C57BL/6J mice with a genetic c-kit expression deficit (C57BL/6J-Kit^W-sh^ mice) and in the wt C57BL/6J controls. However, the Kit^W-sh^ mutant mice have very low numbers of CD117+ ECs (here 1% of total lin−CD31+CD105+ ECs). Typical results from FACS analysis of lung ECs are shown. Histograms indicate the percentage of CD117+ cells, control IgG labeling and gatings are also shown. (B) lin−CD31+CD105+ ECs from kit deficient Kit^W-sh^ mutant mice contain abnormally low levels of endothelial CFCs in comparison to wt mice (*p*<0.0001, the Mann-Whitney test). The horizontal lines indicate 10th, 25th, 50th (median), 75th, and 90th percentiles. The results of 12 independent experiments, each performed in duplicate, are shown. The Mann-Whitney test was used to compare the groups. (C) When syngeneic B16 melanoma tumors were implanted to kit deficient Kit^W-sh^ mutant mice, a highly significant impairment of tumor angiogenesis was observed (*p* = 0.0006; *n* = 7 for each group). vWF/DAPI stains are also shown. The tumor vasculature in kit deficient Kit^W-sh^ mutant mice contained a significantly diminished number of proliferating ECs (*p* = 0.01; *n* = 7 for each group). The percentiles of mean percentages of proliferating (ki-67+) ECs are shown. ki-67/CD31/DAPI stains are also shown. The Mann-Whitney test was used to compare the groups. Scale bars, 100 µm. (D) A highly significant retardation of tumor growth was observed in the kit deficient Kit^W-sh^ mice. *p<0.01; **p<0.001; ***p<0.0001; *n* = 17 for each group.

Mutant C57BL/6J mice with a genetic KitW-sh deficit in c-kit expression were used to study the functional role of CD117 in EC colony-formation and in angiogenesis in vivo. In many adult stem cell types, CD117 plays an important role in stem cell survival and proliferation [27]. The KitW-sh mutation disrupts 5′ regulatory sequences of the c-kit gene [44] and influences c-kit expression in a tissue specific manner affecting melanoblast survival and density [45]. Additionally, c-kit expression is abolished in mast cells, and the KitW-sh mutant mice have a mast cell deficit [46]. However, the KitW-sh homozygotes are healthy and fertile, and produce normal litters [47]. We found that kit deficient C57BL/6J-Kit^W-sh^ mice have much lower numbers of CD117-expressing ECs than the wt control mice (mean 1.5%, SD±0.71 of lin−CD31+CD105+ lung ECs, *n* = 3; Figure 7A). In agreement, isolated ECs from Kit^W-sh^ mice had lower numbers of endothelial CFCs (mean <0.02%, SD±0.012; *p*<0.0001; Figure 7B) than ECs from wt mice. In vivo, Kit^W-sh^ mutation resulted in impaired tumor angiogenesis and retardation of tumor growth (Figure 7C and 7D). Blood vessel densities from kit deficient mice inoculated with syngeneic B16 melanomas were less than half of those from wt C57BL/6J mice (mean vessel count per field 14.7, SD±1.5 versus 34.0, SD±3.8; *p* = 0.006). Correspondingly, the tumor vasculature in the kit deficient mice contained a significantly diminished number of proliferating ECs (mean 17.3%, SD±5.3 versus 25.7%, SD±4.6; *p* = 0.01; Figure 7C).

Importantly, B16 melanoma tumor growth was very significantly inhibited in the kit deficient mice (Figure 7D). To dissect the possible effect from the kit deficient hematopoietic system on tumor growth, wt mice were subjected to total myeloablation and reconstituted with kit deficient KitW-sh mutant BM or with wt BM. When B16 melanomas were later, after the reconstitution of the hematopoietic system from the BM transplant, implanted to mice, no differences in tumor growth were observed between the two groups, regardless of whether the hosts had received a wt (*n* = 14) or a kit deficient BM transplant (*n* = 13; *p*>0.1; Figure S5). Thus, the retardation in tumor angiogenesis and cancer growth we observed in C57BL/6J-Kit^W-sh^ mutant mice is not an effect of the c-kit expression deficit in the hematopoietic system.

## Discussion

**Figure 8:**
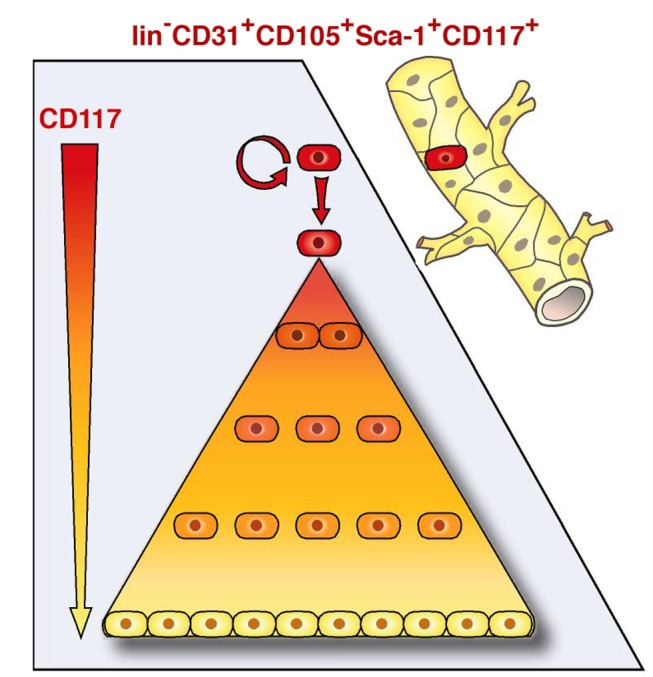
The present results provide evidence for adult endothelial stem cell hierarchy. The results provide evidence for the existence of a rare self-renewing adult VESC that resides at the blood vessel wall endothelium. VESCs are a small subpopulation within vessel wall CD117+ ECs capable of undergoing clonal expansion while other ECs have a very limited proliferative capacity.

While infrequent proliferative cells with endothelial characteristics have been observed circulating in the peripheral blood [22]–[26], genetic fate mapping has suggested that the origin of endothelial stem/progenitor cells involved in adult angiogenesis must be local tissue resident cells [8]. Our present results provide evidence for adult endothelial stem cell hierarchy and the existence of a rare self-renewing CD117+ adult VESC that resides at the blood vessel wall endothelium (Figure 8). In all the divergent assay types we utilized to estimate the frequency of CFCs in different adult EC populations, their occurrence was always only a few CFCs per a thousand total ECs. The CD117+ EC fraction is greatly enriched and the CD117− EC fraction is greatly depleted in colony-forming ECs. Similarly, only CD117+ ECs were able to form blood vessels when transplanted in vivo. In Kit^W-sh^ mice where CD117 expression is deficient in a tissue specific manner, the number of CD117-expressing ECs and endothelial CFCs were significantly reduced, and EC proliferation and angiogenesis were impaired. This observed defective EC phenotype is not unlike the already reported deficiency in melanocyte and mast cell densities in these c-kit mutant mice [45],[46]. Taken together, our present results suggest that the ability to proliferate is not a stochastic property of ECs, and that the proliferative potential of ECs is hierarchically organized [21], with different EC subpopulations discriminated by their clonogenic potential. Our present results identify a population of EC progenitors with a high proliferative and clonogenic potential that is a small subset within CD117+ ECs (Figure 8).

While a subset of ECs had a high capacity to produce endothelial daughter cells in various experimental settings in vitro and in vitro, we observed no evidence of transdifferentiation of other cell types to ECs. The possibility that endothelial colony-forming activity might originate from hematopoietic cells was particularly carefully studied, but this was not observed in the experiments. The result is in agreement with earlier reports suggesting that adult stem/progenitor cells are lineage restricted, and that adult blood vessel endothelium does not originate from hematopoietic cells [8],[11],[12],[23],[48]–[59]. However, the present results cannot directly exclude the possible existence of other cell populations capable of endothelial growth. Colony formation assays are admittedly artificial and do not always necessarily represent what actually occurs in vivo.

Additionally, end differentiated mature vascular wall ECs that normally have very limited proliferative capacity might reversibly acquire more stem cell-like characteristics in vivo in angiogenic situations (including human cancer), maybe for example by turning on CD117 expression (Figure 4B and 4C). Indeed, it has been reported that c-kit expression occurs in a subset of angiosarcomas, probably representing oncofetal expression, i.e., reversion of the tumor cell phenotype to that of fetal ECs that may normally show c-kit expression [60]. Thus, angiogenesis might in certain situations be driven also by abundant ECs that can reversibly acquire stem cell-like characteristics and proliferative potential.

Discovery of additional cell-surface markers should allow more efficient identification and isolation of VESCs than we have currently achieved here. The daughter cells produced by VESCs could compensate for cell loss during normal lifelong cellular turnover, and be responsible for the generation of novel neoangiogenic ECs in angiogenic situations. Together, the present results suggest the possibility of cell-based therapies for cardiovascular repair using isolated, highly enriched VESCs to restore tissue vascularization [61],[62]. VESCs are also a cellular target for future—and also present— therapies that aim to restrain angiogenesis by inhibiting endothelial-cell proliferation. Therefore, stem and progenitor cells for vascular ECs and the molecular machinery governing their activation and functions are novel cellular and molecular targets for therapeutic approaches to inhibit excessive angiogenesis and to block cancer growth [4],[62].

## Materials and Methods

### Isolation of Mouse EC Populations

Mouse lung ECs were isolated from lungs and other indicated tissues dissected from adult wt C57BL/6J mice (Scanbur AB), GFP-tagged transgenic C57BL/6-Tg(ACTB-EGFP)1Osb/J mice, C57BL/6J-Kdrtm1Jrt; lacZ mice [40], FVB/N-Tg(TIE2-lacZ)182Sato/J mice [41], or C57BL/6J-Kit^W-sh^ mice (all from The Jackson Laboratory). Mouse lung ECs were isolated from lungs dissected from adult mice. Mice were anesthetized with Rompun vet (Bayer) and Ketaminol vet (Intervet). The chest was opened through a midline sternotomy. The left ventricle was identified and the ventricular cavity was entered through the apex with a 27-gauge needle. The right ventricle was identified and an incision was made in the free wall to exsanguinate the animal and to allow the excess perfusate to exit the vascular space. The animal was perfused with 20 ml of PBS at approximately 10 ml/min. After being killed, the lungs were collected, fat tissue was removed, and lung tissue was minced manually. The tissue fragments were then digested with DMEM medium containing Dispase II (0.8 U/ml, Roche), collagenase H (1 mg/ml, Roche), Pen/Strep (100 U/100 g/ml), 2% FCS, 2 mM glutamine at 37°C for 1 h after which the suspension was homogenized by pipetting. The homogenate was filtered through a 100-µm nylon mesh filcon (BD Biosciences) and subsequently through a 30-µm nylon mesh falcon (BD Biosciences) and pelleted by centrifugation (300*g* for 6 min). Erythrocytes were lysed in lysis buffer containing 10 mM KHCO3, 155 mM NH4Cl, 0.1 mM EDTA, (pH 7.5) at room temperature for 2 min. The cell pellets were resuspended in DMEM medium containing Pen/Strep (100 U/100 g/ml), 2% FCS, and 2 mM glutamine. For lineage depletion, Mouse Lineage Cell Depletion Kit (Miltenyi Biotec) was used according to manufacturer's instructions. Subsequently, the cells were incubated with anti-mouse CD16/CD32 blocker (BD Pharmingen) according to the manufacturer's instructions to reduce FcγII/III receptor-mediated antibody binding stained for FACS. The antibodies used included APC anti-mouse CD31 (BD Pharmingen) and PE anti-mouse CD105 (eBioscience), V450 Sca-1 (BD Biosciences), and PE-Cy7 CD117 (eBioscience). Cells were analyzed and sorted using BD FACSAria flow cytometer and cell sorting system (BD Biosciences). Compensation adjustments were performed with single color positive controls. Dead cells and cell debris were excluded by gating the population according to the forward and side light scatters. The positive cells were compared the cells positive in stainings with IgG isotype controls (BD Pharmingen). Before each sorting, laser compensations were adjusted automatically with FACSDiva software version 4.1.2 (BD Biosciences). For single cell sorting, automated cell deposition unit (ACDU) in FACSAria was used for single cell sorting of populations onto 96 multi-well plates. The presence of a single GFP+ cell per well was checked under fluorescence microscope after sorting. A carrier population of 2,500 freshly isolated wt (GFP−) lin−CD31+CD105+Sca-1+CD117+ cells per well was plated together with the GFP+ single cells. The carrier population was used because the single cells were not expected to be able to survive completely alone in the well. For some of the preliminary in vitro assays, CD31+CD105+ ECs were isolated using anti-fluorochrome multisort kit (Miltenyi Biotec) according to the instructions of the manufacturer. The Provincial State Office of Southern Finland approved all the animal experiments.

### In Vitro EC Colony Assays

Freshly isolated ECs were plated in duplicate in 1 ml of 0.8% methylcellulose containing 15% FCS, 1% L-glutamine, 1% BSA, 10^−4^ mM 2-mercaptoethanol, 0.2 mg/ml human transferring, 0.01 mg/ml rh insulin (all from Stemcell Technologies) supplemented with 100 ng/ml recombinant murine VEGF (Invitrogen). Colonies were studied and counted at days 7, 10, and 14. Scoring of colonies was performed with an inverted microscope. Colonies containing 15 or more cells at day 7 were counted. In preliminary experiments the isolated cell populations were plated at various plating densities, and an ideal plating density for each population was determined.

### In Vitro Hematopoietic Cell Colony Assays

BM cells were collected by flushing femurs and tibias of the donor mice with 29-gauge needle into DMEM (Invitrogen) supplemented with 2-mM L-glutamin, 100 units/ml penicillin, and 100 g/ml streptomycin (Invitrogen). CD45+ BM cells were selected using CD45 microbeads, mouse (Miltenyi Biotec) according to manufacturer's instructions. Lineage depleted cells were selected using Mouse Lineage Cell Depletion Kit (Miltenyi Biotec) according to manufacturer's instructions. Freshly isolated cells were plated in duplicated in 1 ml of MethoCult GF M3534 medium (contains rm stem cell factor, rm IL-3, and rh IL-6; Stemcell technologies) supplemented with 10 ng/ml GM-CSF (Invitrogen) and 10 ng/ml M-CSF (Invitrogen). Different plating densities ranging from 1,000 to 100,000 cells per plate were studied. Colonies were studied and counted at days 7, 10, and 14.

### lacZ Stainings

Freshly isolated lung ECs from transgenic lacZ-carrying reporter mouse strains or from wt mice (negative controls) were plated in 1 ml of 0.8% methycellulose containing 15% FCS, 1% L-glutamine, 1% BSA, 10^−4^ mM 2-mercaptoethanol, 0.2 mg/ml human transferin, 0.01 mg/ml rh insulin (all from StemCell technologies) supplemented with 100 ng/ml rm VEGF (Invitrogen). After 7 d, methycellulose was washed by PBS. Colonies were stained using ImaGene C12FDG lacZ Gene Expression Kit (Invitrogen) according to the manufacturer's instructions. The colonies were studied and photographed with Axiovert 200 inverted epifluorescence microscope (Carl Zeiss) using a Plan-Neofluar 10× objective (NA = 0.3), Zeiss AxioCam HRc color camera and Zeiss AxioVision 3.1 software.

### EC Culture

Freshly isolated ECs were cultured on gelatin coated plates or flasks in IMDM medium containing 15% FCS, 1% L-glutamine, 1% BSA, 10^−4^ mM 2-mercaptoethanol, 0.01 mg/ml rh insulin, 100 ng/ml rm VEGF, 100 ng/ml rm bFGF, and 100 ng/ml rm EGF (all from Invitrogen), and 0.2 mg/ml human transferring (Sigma-Aldrich) at 37°C in a humidified 5% CO_2_ atmosphere. To study cultured passaged ECs, the cells were fixed with 4% PFA, blocked with PBS buffer containing 5% serum (Vector Laboratories), and incubated with the primary antibodies overnight at 4°C and subsequently detected with fluorochrome-conjugated secondary antibodies for 30 min at RT. The primary antibodies used in immunofluorescence were rat anti-mouse VEGFR-2 (BD Pharmingen), rat anti-mouse CD45 (BD Pharmingen), FITC conjugated rat anti-mouse CD14 (BD Pharmingen), rat anti-mouse CD16/32 (BD Pharmingen), Alexa 488 anti-mouse CD115 (BD Pharmingen), rat anti-mouse to F4/80 (Abcam), monoclonal anti-actin, alpha-smooth (Sigma), and FITC conjugated anti-mouse CD34 (BD Pharmingen). The secondary antibodies used were Alexa594 anti-rat. The samples were analyzed and photographed with an Axioplan 2 upright epifluorescence microscope using 20× (NA = 0.50) and 40× (NA = 0.75) Plan-Neofluar objectives, AxioCam Hrc camera 14-bit grayscale CCD camera, and Axiovision 4.3 software (Carl Zeiss).

### Cell-Surface Marker Analyses by FACS

For MS-1 EC line (ATCC number 2279; a kind gift from Jack L. Arbiser, Emory University School of Medicine, Atlanta), and cultured passage 1 and 24 ECs, the cultures were washed with PBS, detached by 1 mM EDTA at 37°C for 10 min, and cell pellets were collected. The studied cells were incubated with anti-mouse CD16/CD32 blocker (BD Pharmingen) according to the manufacturer's instructions to reduce FcγII/III receptor-mediated antibody binding stained for FACS. The antibodies used inculed APC anti-mouse CD31 (BD Pharmingen), PE anti-mouse CD105 (BD Pharmingen), V450 anti-mouse Sca-1 (BD Pharmingen), PE-Cy7 anti-mouse CD117 (eBioscience), FITC anti-mouse CD45 (BD Pharmingen), FITC anti-mouse CD11b (BD Pharmingen), Alexa 700 anti-mouse VEGFR2 (eBioscience), Alexa 700 anti-mouse CD34 (eBioscience), FITC anti-mouse CD115 (eBioscience), FITC anti-mouse CD14 (eBioscience), FITC anti-mouse F4/80 (eBioscience), Alexa 488 anti-mouse VEGFR-1 (R&D systems), FITC anti-mouse CD104 (Clone 346-11A, Abcam), FITC anti-mouse CD106 (BD Pharmingen), FITC anti-mouse CD184 (BD Pharmingen), Alexa 488 anti-mouse CD144 (eBioscience), and FITC anti-mouse alpha smooth muscle actin (Abcam). Cells were analyzed and sorted using BD FACSAria flow cytometer and cell sorting system (BD Biosciences). Compensation adjustments were performed with single color positive controls. Dead cells and cell debris were excluded by gating the population according to the forward and side light scatters. The positive cells were compared the cells positive in stainings with IgG isotype controls (BD Pharmingen). A minimum of 30,000 total events was always analyzed. Before each analysis, laser compensations were adjusted automatically with FACSDiva software (BD Biosciences). Mean fluorescence intensity (MFI) values are presented as the mean ± SD, calculated from multiple mice over three experiments. Statistical significance was assessed by the Student's *t* test, and *p* values<0.05 were considered significant.

### Total RNA Purification and Real-Time Quantitative RT-PCR

Total RNA from freshly isolated mouse lung ECs was extracted using RNeasy Mini kit (Qiagen) according to manufacturer's instruction. Sample integrity was analyzed with Agilent 2100 BioAnalyzer to demonstrate consistent quality. Real-time quantitative RT-PCR analyses (RT2 qPCR Primer Assays) were performed by SABiosciences-QIAGEN using the SYBR Green real-time PCR detection method. To normalize the data the Ct value of the housekeeping gene GAPDH was subtracted from the value of the gene of interest. The 2∧(-delta Ct) was used to calculate the normalized relative quantity in order to compare the relative quantification of the gene of interest.

### In Vivo Cell Transplantations

For transplanting the progeny of a single EC, GFP-tagged cells were isolated from transgenic C57BL/6-Tg(ACTB-EGFP)1Osb/J mice, and the single cell suspension was plated in adherent colony-forming assays at one CFC per plate. The colonies were expanded for 12 d in adherent matrix. Subsequently, the plates and the colonies were studied under Axiovert 200 inverted epifluorescence microscope (Carl Zeiss) using both bright field and eGFP channels. The plates that contained a single clonal colony per plate after the expansion were utilized in cell transplantations. The colonies were under Olympus CKX31 inverted microscope and were manually collected from the dish bottom (where they grow adherent to the plastic) by scraping and carefully pipetting using a micropipette. The cells were then resuspended in PBS, and mixed with 200 µl of matrigel (Basement Membrane Matrix; BD Pharmingen) supplemented with VEGF (100 ng/ml; Invitrogen) and bFGF (10 ng/ml; Invitrogen), and injected subcutaneously into wt C57BL/6J mice. After 2–3 wk, the mice were killed and the vasculature of the plugs analyzed. In some experiments, defined numbers of freshly isolated GFP-tagged cells were used. In repeated isolations and serial transplantations in vivo, C57BL/6J mice were inoculated with syngeneic B16 melanoma cells (2×10^6^ B16 cells in 200 µl) mixed with 15 CFUs of GFP-tagged CD31+CD105+ ECs. After 2 wk, the tumor was excised and processed to single cell suspension that was lineage depleted using Mouse Lineage Cell Depletion Kit (Miltenyi Biotec) according to manufacturer's instructions. The lineage depleted cells (containing B16 cells and 10,000 GFP-tagged ECs per tumor) were then inoculated to a new host. The isolation and retransplantation was repeated every 2 wk. Part of the tumors in each generation was processed for tissue analyses.

### Induction of Angiogenesis by Syngeneic B16 Melanoma Tumors or Matrigel Plugs

The B16-F1 melanoma cell line (ATCC number 6323) was maintained in DMEM supplemented with 2 mM L-glutamine, Pen/Strep (100 U/100 g/ml), and 10% FBS (PromoCell). The mice were injected subcutaneously with B16 cells (2×10^6^ cells in 200 µl), and the tumors were let to grow for 10–20 d. Matrigel plugs (400 µl per injection Basement Membrane Matrix; BD Pharmingen) supplemented with recombinant murine VEGF (100 ng/ml; Invitrogen) were injected subcutaneously to the back of the mouse. The plugs were excised and processed for tissue analyses at 2–3 wk after injection. In some experiments, GFP+ cell populations isolated from transgenic C57BL/6-Tg(ACTB-EGFP)1Osb/J mice were mixed with B16 cells or matrigel prior to injection. In experiments where tumor growth rate was studied, 2×10^6^ B16 cells suspended in 200 µl growth factor reduced matrigel were injected subcutaneously. The tumors were measured every other day using a vernier caliper. Tumor volume was determined using the Pi/6×L×W×W formula with L as the longest diameter and W the diameter at the position perpendicular to L.

### Immunohistochemistry and Whole Mounts

The primary antibodies used in immunoflurescence were rat anti-mouse CD31/PECAM-1 (BD Pharmingen) rat anti-mouse CD105/endoglin (BD Pharmingen), rat anti-mouse VEGFR-2 (BD Pharmingen), rabbit anti-mouse/human von Willebrand Factor (vWF; DAKO), rat anti-mouse Sca-1 (BD Pharmingen), rabbit polyclonal anti-mouse VE Cadherin (Abcam), rabbit anti-ZO-1 (N-term) (Invitrogen), and goat anti-mouse CD117 (R&D Systems). In some stainings, rat anti-mouse CD45 (BD Pharmingen) and rabbit polyclonal anti-β-galactosidase (Chemicon International) were used as negative controls. The secondary antibodies used were Alexa594 anti-rat, Alexa594 anti-rabbit, Alexa633 anti-rat, Alexa633 anti-rabbit, Alexa 594 anti-goat, Alexa 647 anti-goat, Alexa 647 anti-rabbit, Alexa488 anti-rat, Alexa 488 anti-rabbit (all from Molecular Probes). For staining of whole mounts, all samples were fixed in 4% PFA, blocked with PBS buffer containing 5% serum (Vector Laboratories), 0.2% BSA, 0.09% Na-Azide, 0.2% BSA, and 0.3% Triton-X (Sigma-Aldrich), and incubated with the primary antibodies for 2 d at room temperature. Auto-fluorescent cartilage was removed from the ears before fixing. The samples were washed and incubated with fluorochrome conjugated secondary antibodies overnight at room temperature. Finally, the plugs were sliced, the ears were flattened, and the samples were mounted with antifading medium (Vectashield; Vector Laboratories). For fluorescent immunohistochemistry of cryosections, samples were fixed for 1 h with 2% PFA and incubated in 20% sucrose/PBS overnight. After the cryopreservation, tissues were embedded in OCT compound (Tissue-Tek; Sakura Finetek Europe) and frozen at −70°C. Sections (8–80 µm) were stained with the primary antibodies overnight at 4°C and subsequently detected with fluorochrome-conjugated secondary antibodies for 30 min at room temperature. Finally, the sections were mounted with anti-fading medium (Vectashield). To verify that detected blood vessels were actually functional vessels that were connected to the blood circulation, the mice were perfused with fluorescein-labeled microspheres essentially as described earlier [42]. Fluorescent carboxylate-modified microspheres, 0.2-µm, red fluorescent (580/605) (FluoSpheres, Molecular Probes) were used diluted 1∶6 with PBS. Mice were anesthetized with Rompun vet (Bayer) and Ketaminol vet (Intervet). Tridil (nitroglycerin, Orion Oyi, Espoo, Finland) was included in the anesthesia mixture at 50 mg/ml to allow maximal vasodilation of the peripheral vasculature. The chest was opened through a midline sternotomy. The left ventricle was identified and the ventricular cavity was entered through the apex with a 27-gauge needle. The right ventricle was identified and an incision was made in the free wall to exsanguinate the animal and to allow the excess perfusate to exit the vascular space. The animal was perfused with 4–6 ml of PBS at approximately 10 ml/min and then with the fluorescent microspheres. For staining of colonies grown in colony assays, methylcellulose was washed with PBS and cells were fixed in 4% PFA; blocked with PBS buffer containing 5% serum (Vector Laboratories), 0.2% BSA, 0.09% Na-Azide, 0.2% BSA, and 0.3% Triton-X (Sigma-Aldrich), and incubated with the primary antibodies for overnight at 4°C and subsequently detected with fluorochrome-conjugated secondary antibodies for 1 h at room temperature. The samples were analyzed with a Zeiss LSM510 laser scanning confocal microscope (Carl Zeiss; LSM 5 Software version 3.2) using multichannel (sequential) scanning in frame mode using a 40× (NA = 1.3) Plan-Neofluar and a 63× (NA = 1.4) Plan-Apochromat oil immersion objectives. Single XY-scans typically had an optical slice thickness of 0.9 µm or less. Additionally, the samples were analyzed and photographed with an Axioplan 2 upright epifluorescence microscope using 5× (NA = 0.15), 10× (NA = 0.3), 20× (NA = 0.50), and 40× (NA = 0.75) Plan-Neofluar objectives, AxioCam Hrc camera 14-bit grayscale CCD camera, and Axiovision 4.3 software (Carl Zeiss). 3D reconstitutions were created with ImageJ 1.42q using Volume Viewer plugin in Volume II mode. For bright field immunohistochemistry, 7-µm cryosections were air-dried and fixed in cold acetone for 10 min and processed essentially as described earlier [63]. The sections were first treated with 0.3% H_2_O_2_ in methanol to block endogenous peroxidase. After endogenous peroxidase blocking, the sections were rehydrated in PBS for 5 min and incubated in 5% normal serum at room temperature for 30 min, and incubated with rat anti-mouse CD117 (R&D Systems) at 4°C overnight. Negative controls were performed by omitting the primary of secondary antibodies or by using irrelevant primary antibodies of the same isotype. A subsequent incubation for 30 min with biotinylated anti-rat IgG was followed by a 30-min incubation using ABC reagents of the Vectastain Elite Rat IgG ABC Kit (Vector Laboratories) and a 10-min incubation with Chromogen Immpact DAB (Vector Laboratories). The slides were counterstained with Mayer's hematoxylin (DAKO) and mounted using VectaMount AQ medium (Vector Laboratories), and studied and photographed with Leica DM LB microscope (Leica Microsystems GmbH) using C Plan 63× objective (NA = 0.75) and Olympus DP50 color camera (Olympus Corporation). Tumor vessel density was determined from the peri- and intratumoral area in four to six fields (×100) of CD31 stainings of the tissue sections in areas with the highest vascularity (hot spots) essentially as described by Folkman and coworkers [64], and the results were recorded as the sum of all vessel counts per field. Only structures that morphologically appeared as vascular and stained with the immunomarker were taken into account. The scoring was performed blinded to avoid bias. The final score for each tumor was determined as a mean of the values determined from intra- and/or peritumoral vessel densities. For assessment of the proliferating fraction of tumor ECs in B16 tumors, proliferating (ki-67+) EC (CD31+) hotspots were identified by scanning the sections at low magnification by using 10× (NA = 0.3) plan-Neofluar objective and Axioplan2 epifluorescence microscope (Carl Zeiss). Micrographs were then taken using 40× (NA = 1.3) oil objective in four to 18 fields and Axiovision 4.3 software. Total CD31+ ECs and CD31+/ki–67+ ECs within the micrograph area (0.097 mm^2^) were manually calculated using Image J (NIH Image) via cell counter plug-in. The results were recorded as mean for each tumor. The scoring was performed blinded to avoid bias.

### Human Tumor Tissue Samples

Paraffin-embedded human tumor tissue samples from patients with histologically diagnosed malignant melanoma (*n* = 4) or malignant breast cancer (*n* = 10) were drawn from the archives of the Department of Pathology, University of Helsinki. Heat-induced epitope retrieval was performed by heating the deparaffinized tissue sections in a buffered citric acid solution (Dako) for 15 min. The sections were incubated for 30 min in 0.3% hydrogen peroxidase in methanol at room temperature and for 30 min in 5% normal horse serum at room RT. CD117 expression was determined using mouse anti-human CD117 Mab (MS-289-P; NeoMarkers) diluted 1∶100 incubated overnight at 4°C at a dilution of 1∶100. A subsequent incubation for 30 min in biotinylated anti-mouse serum was followed by a 30-min incubation using reagents of the Vectastain Elite ABC kit (Vector Laboratories). Peroxidase activity was developed using DAB (Vector) for 10 min. Finally, the sections were stained with hematoxylin for 30 sec and mounted with VectaMount AQ (Vector). Negative controls were performed by omitting the primary antibody or by using irrelevant primary antibodies.

### Syngeneic BM Transplantations

BM cells were collected by flushing femurs and tibias of the donor mice with 29G needle into DMEM (Invitrogen Corporation) supplemented with 2 mM L-glutamin, penicillin (100 U/ml) and streptomycin (100 µg/ml) (Sigma-Aldrich). Unselected BM cells (7×10^6^) were transplanted into corresponding syngeneic wt mice via tail vein injection. The recipients were irradiated 1 d prior to transplantation by a lethal dosage of 9.1 Gy. Mice were used for experiments 5–8 wk after transplantation.

## Supporting Information

Figure S1 **Cell-surface marker analyses of cultured lin−CD31+CD105+ ECs at passage 24.** (A) Long term 2-D EC cultures originating from individual lin−CD31+CD105+ CFCs that were picked up from colony assays from isolated lin−CD31+CD105+ mouse lung ECs were analyzed by immunofluorescence microscopy. The nuclei are stained with DAPI (blue) to recognize individual cells. Scale bars, 50 µm. (B) The individual cultures were later transferred to T75 flasks and some of them were propagated for over 20 passages. Micrographs of a confluent monolayer at passage 21 are shown. Scale bars, 200 µm.
(TIF)

Figure S2 **Real-time quantitative RT-PCR analyses of freshly sorted lung lin−CD31+CD105+Sca1+CD117+ cells.** To control the purity of the isolated ECs and make certain that hematopoietic cells were properly removed after lineage depletion and subsequent FACS sorting of the CD31+CD105+Sca1+CD117+ cells, the freshly isolated lung cells were assayed using real-time quantitative RT-PCR for mRNAs of endothelial or hematopoietic markers. To normalize the data the Ct value of the housekeeping gene GAPDH was subtracted from the value of the gene of interest. The 2∧(−delta Ct) was used to calculate the normalized relative quantity in order to compare the relative quantification of the gene of interest. Four independent experiments with one mouse per group were performed. mRNA expression profile from the isolated ECs corresponds to what is expected from ECs. Note that ECs normally express low levels of various “hematopoietic” markers including CD14 [36]–[39], CD16 [36], and CD45 [38],[65].
(TIF)

Figure S3 **CD117+ ECs are detected both in arteries and veins.** Immunostaining for CD117 normal lung tissue. CD117+ expressing ECs are indicated with arrows. Note the red blood cells at the vessel lumina. Scale bars, 10 µm. (A) An artery is shown. (B) A vein.
(TIF)

Figure S4 **Hematopoietic cells from the BM produce classical hematopoietic colonies while lin−CD31+CD105+Sca1+CD117+ ECs isolated from the lung produce completely dissimilar EC colonies.** (A) CD45-enriched hematopoietic cells from the mouse BM produce classical hematopoietic colonies in standardized murine hematopoietic CFC assays. BM CD45+ cells were isolated by standard by immunomagnetic separation. Scale bar 100 µm. (B) On the same assay system, lin−CD31+CD105+Sca1+CD117+ ECs isolated from the lung produce EC colonies that are completely dissimilar from hematopoietic colonies. Note the classical EC confluent cobblestone monolayer morphology of the colony on the left, and the also classical “activated” 3D sprouting EC appearance of the other two EC colonies. Scale bar 100 µm. (C) Fluorescence-based detection of β-galactosidase activity in colonies formed from lin−CD31+CD105+Sca1+CD117+ cells isolated from wt C57BL/6J mouse (a negative control for the lacZ reporter gene detection).
(TIF)

Figure S5 **Mice with wt or kit defective BM have equal B16 melanoma tumor growth rates.** To dissect the possible effect from the kit deficient hematopoietic system to tumor growth, wt C57BL/6J mice were subjected to total myeloablation by a lethal dose of whole body gamma irradiation, and reconstituted with equal amounts (7×10^6^ cells) of unselected BM cells from kit deficient C57BL/6J-Kit^W-sh^ or wt C57BL/6J mice. After a recovery of a minimum of 5 wk, syngeneic B16 melanomas were implanted. The experiment was repeated three times, and a total of 14 mice with a wt BM and 13 mice with a kit deficient BM were analyzed. No differences in tumor growth were observed between the groups, regardless whether the mice had received a wt or a kit deficient BM (*p*>0.1 for all comparisons, the Mann-Whitney test).
(TIF)

Table S1 **Summary of cell-surface marker expression of EC monolayers originating from single CFCs.** Surface marker expression was analyzed using FACS from 1st and 24th passage of monolayers cultured from individual colonies picked up from colony assays, and from murine EC line MS-1. Three independent samples of each sample type were analyzed. Scoring: **+++**, 50%–100%; **++**, 10%–50%; **+**, 2%–10%; **+/−**, 0.5%–2%; **−**<0.5%.
(DOC)

## Abbreviations

BM: bone marrow
CFC: colony-forming cell
CFU: colony-forming unit
EC: endothelial cell
FACS: fluorescence activated cell sorter
GFP: green fluorescent protein
HSC: hematopoietic stem cell
RT: reverse transcription
SD: standard deviation
VE-cadherin
vascular endothelial cadherin
VESC: vascular endothelial stem cell
wt: wild type
