# Supplementary figures and images for: Generation of Functional Blood Vessels from a Single c-kit+ Adult Vascular Endothelial Stem Cell
Source: PLoS Biol. 2012 Oct 16;10(10):e1001407. doi: 10.1371/journal.pbio.1001407 (PMC3473016; doi:10.1371/journal.pbio.1001407)

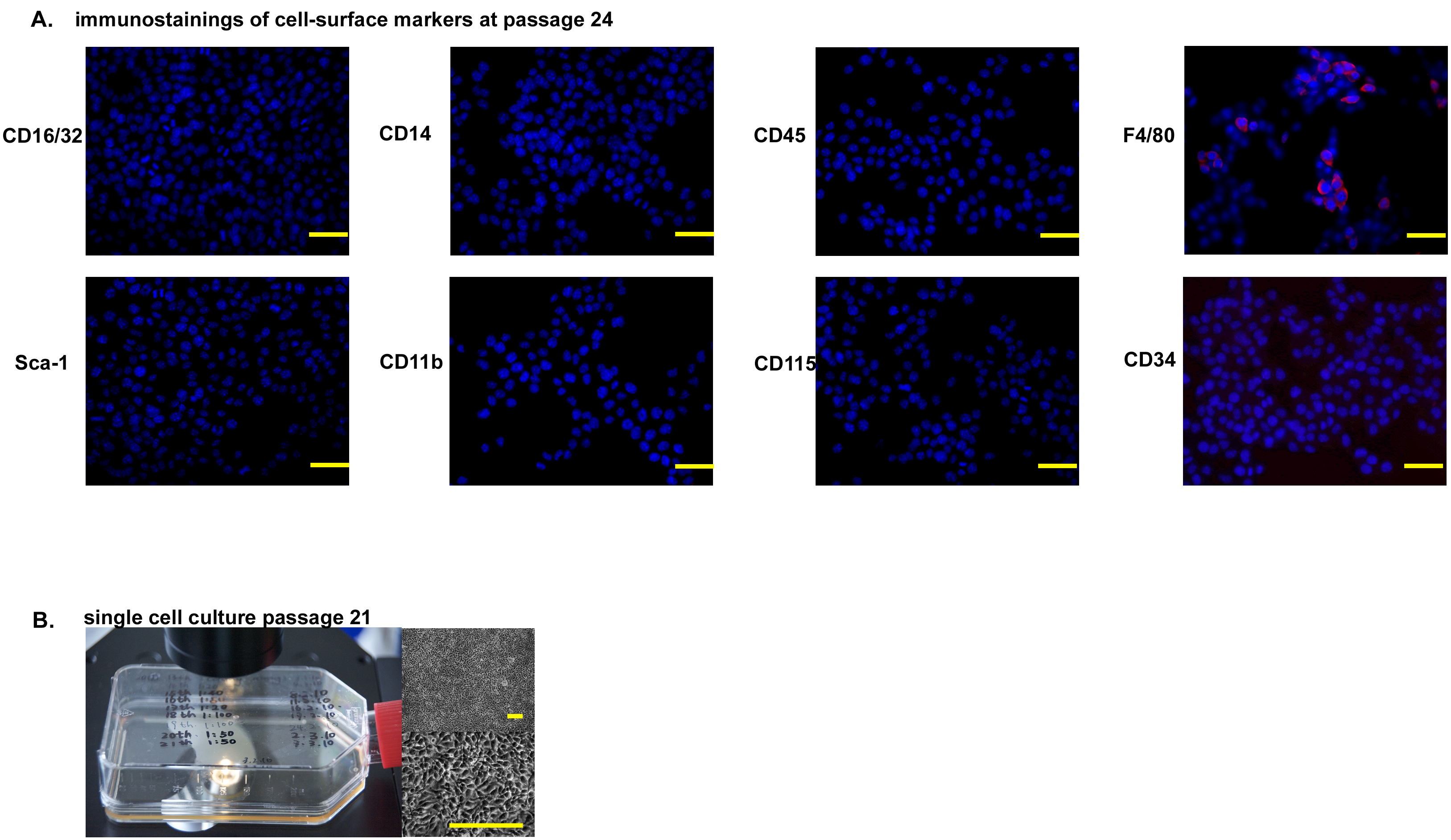

Supplement: Figure S1 — Cell-surface marker analyses of cultured lin−CD31+CD105+ ECs at passage 24. (A) Long term 2-D EC cultures originating from individual lin−CD31+CD105+ CFCs that were picked up from colony assays from isolated lin−CD31+CD105+ mouse lung ECs were analyzed by immunofluorescence microscopy. The nuclei are stained with DAPI (blue) to recognize individual cells. Scale bars, 50 µm. (B) The individual cultures were later transferred to T75 flasks and some of them were propagated for over 20 passages. Micrographs of a confluent monolayer at passage 21 are shown. Scale bars, 200 µm. (TIF) [file pbio.1001407.s001.tif]

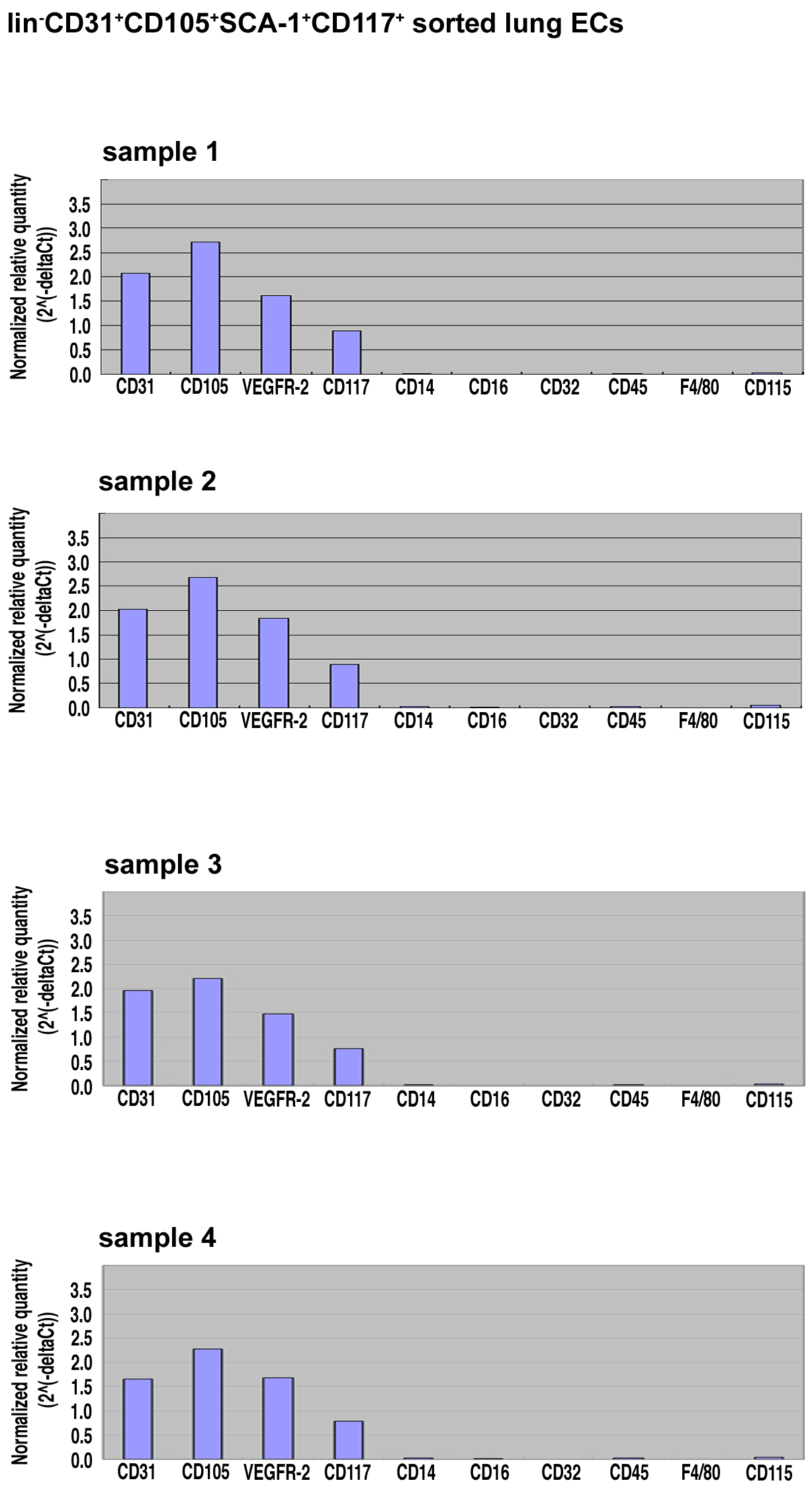

Supplement: Figure S2 — Real-time quantitative RT-PCR analyses of freshly sorted lung lin−CD31+CD105+Sca1+CD117+ cells. To control the purity of the isolated ECs and make certain that hematopoietic cells were properly removed after lineage depletion and subsequent FACS sorting of the CD31+CD105+Sca1+CD117+ cells, the freshly isolated lung cells were assayed using real-time quantitative RT-PCR for mRNAs of endothelial or hematopoietic markers. To normalize the data the Ct value of the housekeeping gene GAPDH was subtracted from the value of the gene of interest. The 2∧(−delta Ct) was used to calculate the normalized relative quantity in order to compare the relative quantification of the gene of interest. Four independent experiments with one mouse per group were performed. mRNA expression profile from the isolated ECs corresponds to what is expected from ECs. Note that ECs normally express low levels of various “hematopoietic” markers including CD14 [36]–[39], CD16 [36], and CD45 [38],[65]. (TIF) [file pbio.1001407.s002.tif]

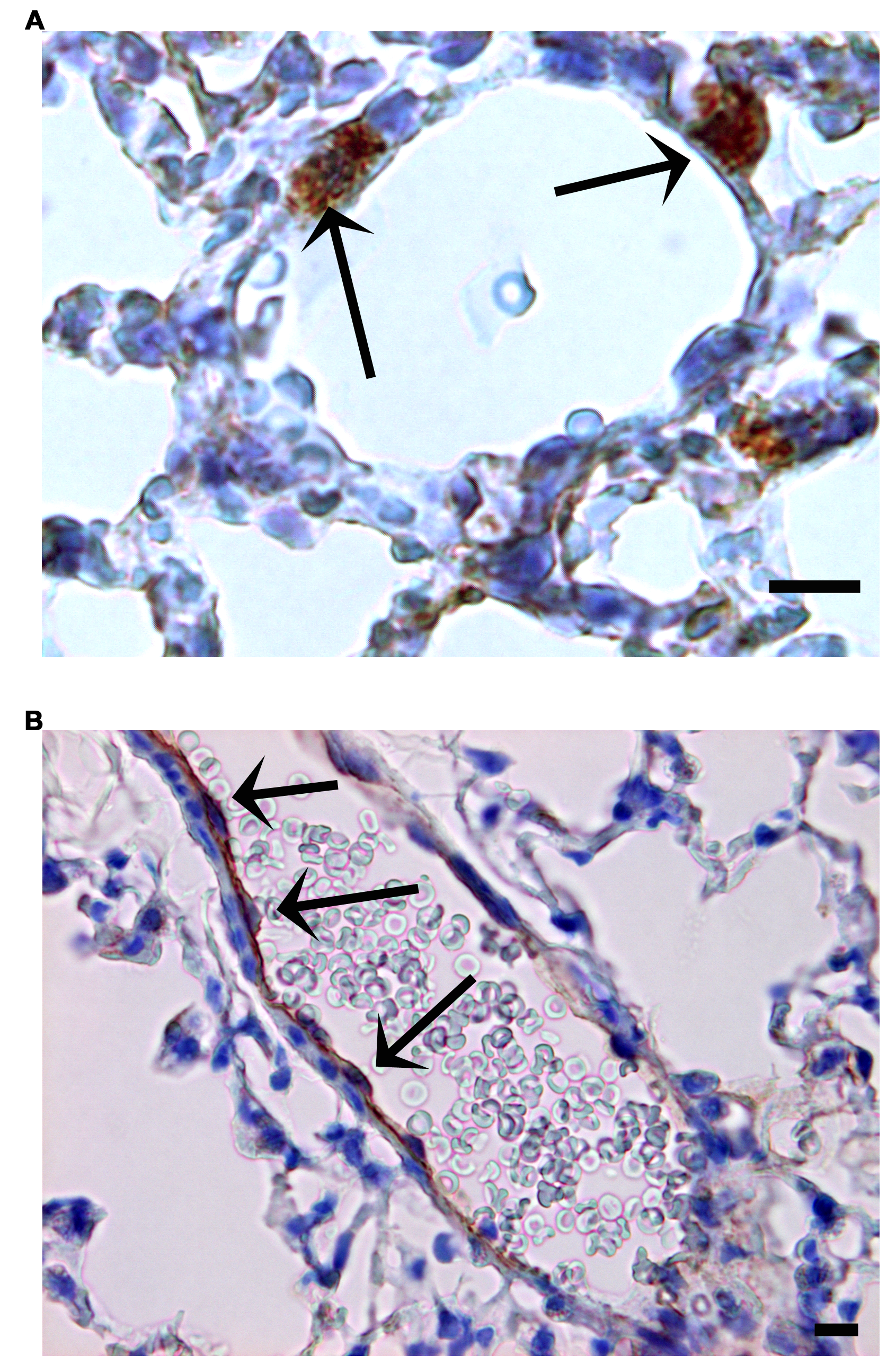

Supplement: Figure S3 — CD117+ ECs are detected both in arteries and veins. Immunostaining for CD117 normal lung tissue. CD117+ expressing ECs are indicated with arrows. Note the red blood cells at the vessel lumina. Scale bars, 10 µm. (A) An artery is shown. (B) A vein. (TIF) [file pbio.1001407.s003.tif]

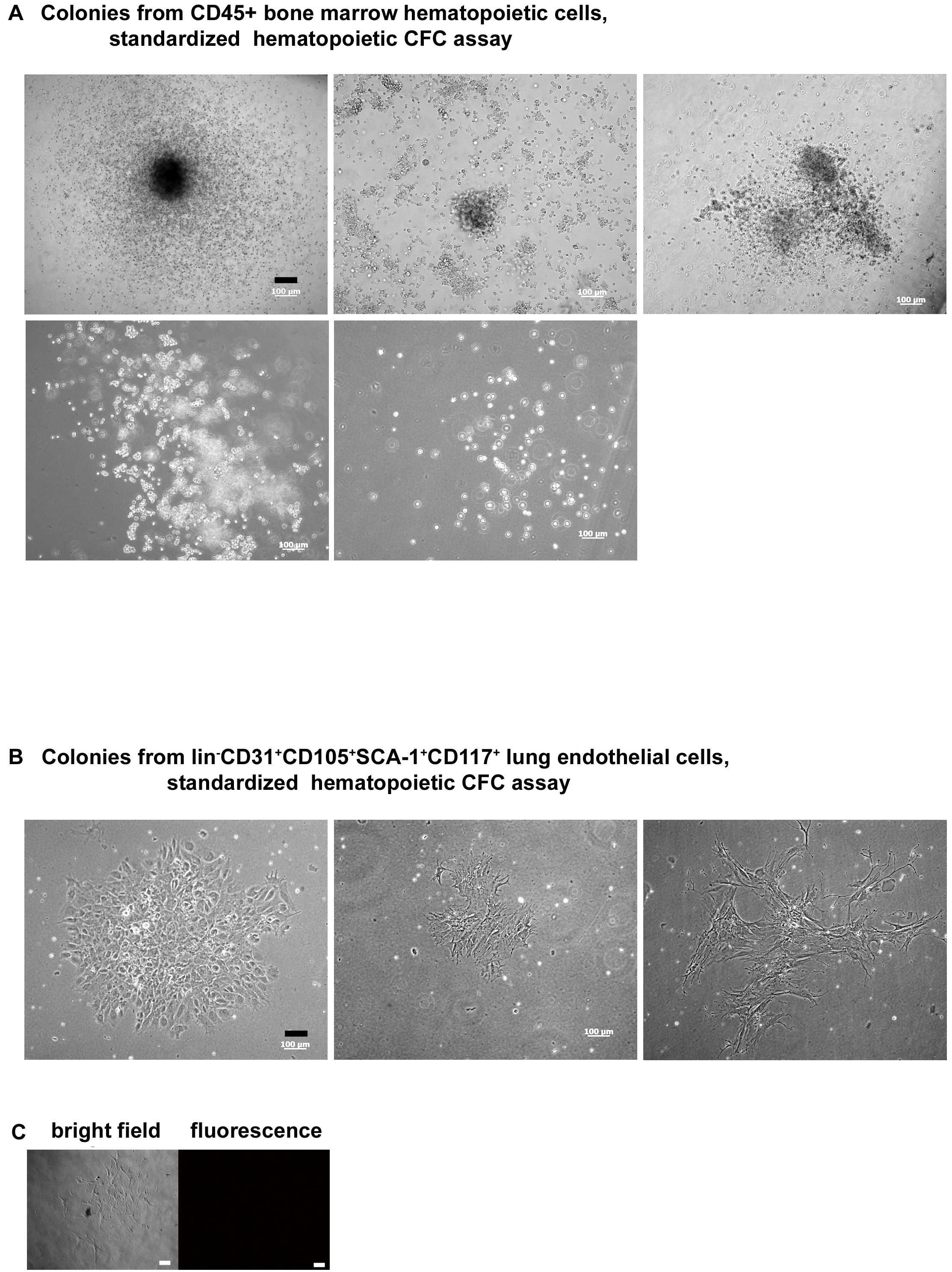

Supplement: Figure S4 — Hematopoietic cells from the BM produce classical hematopoietic colonies while lin−CD31+CD105+Sca1+CD117+ ECs isolated from the lung produce completely dissimilar EC colonies. (A) CD45-enriched hematopoietic cells from the mouse BM produce classical hematopoietic colonies in standardized murine hematopoietic CFC assays. BM CD45+ cells were isolated by standard by immunomagnetic separation. Scale bar 100 µm. (B) On the same assay system, lin−CD31+CD105+Sca1+CD117+ ECs isolated from the lung produce EC colonies that are completely dissimilar from hematopoietic colonies. Note the classical EC confluent cobblestone monolayer morphology of the colony on the left, and the also classical “activated” 3D sprouting EC appearance of the other two EC colonies. Scale bar 100 µm. (C) Fluorescence-based detection of β-galactosidase activity in colonies formed from lin−CD31+CD105+Sca1+CD117+ cells isolated from wt C57BL/6J mouse (a negative control for the lacZ reporter gene detection). (TIF) [file pbio.1001407.s004.tif]

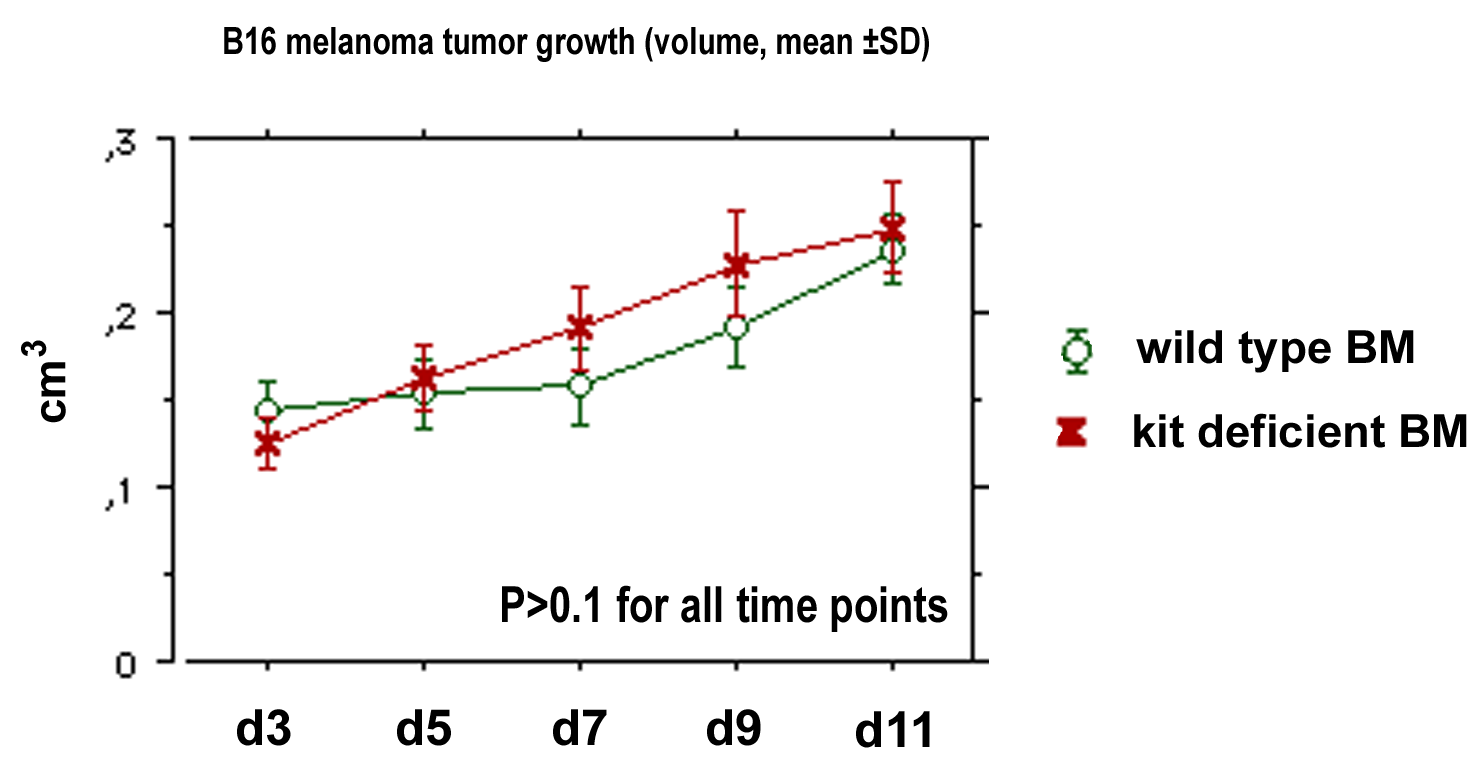

Supplement: Figure S5 — Mice with wt or kit defective BM have equal B16 melanoma tumor growth rates. To dissect the possible effect from the kit deficient hematopoietic system to tumor growth, wt C57BL/6J mice were subjected to total myeloablation by a lethal dose of whole body gamma irradiation, and reconstituted with equal amounts (7×10^6^ cells) of unselected BM cells from kit deficient C57BL/6J-KitW-sh or wt C57BL/6J mice. After a recovery of a minimum of 5 wk, syngeneic B16 melanomas were implanted. The experiment was repeated three times, and a total of 14 mice with a wt BM and 13 mice with a kit deficient BM were analyzed. No differences in tumor growth were observed between the groups, regardless whether the mice had received a wt or a kit deficient BM (p>0.1 for all comparisons, the Mann-Whitney test). (TIF) [file pbio.1001407.s005.tif]
