# Supplementary material for: Generation of Functional Blood Vessels from a Single c-kit+ Adult Vascular Endothelial Stem Cell
Source: PLoS Biol. 2012 Oct 16;10(10):e1001407. doi: 10.1371/journal.pbio.1001407 (PMC3473016; doi:10.1371/journal.pbio.1001407)
Supplement: Table S1 — Summary of cell-surface marker expression of EC monolayers originating from single CFCs. Surface marker expression was analyzed using FACS from 1st and 24th passage of monolayers cultured from individual colonies picked up from colony assays, and from murine EC line MS-1. Three independent samples of each sample type were analyzed. Scoring: +++, 50%–100%; ++, 10%–50%; +, 2%–10%; +/−, 0.5%–2%; −<0.5%. (DOC) [file pbio.1001407.s006.doc]

Table 1

| **Cell-surface marker** | Singe colony monolayer passage 1 | Single colony monolayer passage 24 | MS-1 murine endothelial cell line (control) |
| --- | --- | --- | --- |
| CD31 | +++ | +/- | +++ |
| CD105 | +++ | +++ | ++ |
| Sca-1 | +++ | +/- | +++ |
| CD117 | ++ | + | ++ |
| VEGFR-2 | ++ | + | +++ |
| CD34 | ++ | +/- | +++ |
| CD45 | - | +/- | - |
| CD11b | - | - | - |
| CD14 | +/- | +/- | +/- |
| CD115 | +/- | +/- | - |
| F4/80 | +/- | +/- | - |
| SMA | - | - | - |
